# Exercise Participation and Clinical Characteristics of Desk-Based Workers with Chronic Non-Specific Neck Pain Exposed to Increased Ergonomic Risk: A Cross-Sectional Study

**DOI:** 10.3390/medicina62081602

**Published:** 2026-08-20

**Authors:** Eda Acikalin, Aynur Demirel, Songul Atasavun Uysal

**Affiliations:** Faculty of Physical Therapy and Rehabilitation, Hacettepe University, Ankara 06100, Türkiye; aynur.demirel@hacettepe.edu.tr (A.D.); songula@hacettepe.edu.tr (S.A.U.)

**Keywords:** chronic non-specific neck pain, desk-based workers, exercise participation, ergonomic risk, upper-extremity disability, body awareness, physical activity, RULA

## Abstract

*Background and Objectives:* Chronic non-specific neck pain (CNNP) is common among desk-based workers exposed to increased ergonomic risk and is accompanied by functional impairments. This study examined associations between exercise participation and pain intensity, upper-extremity disability, body awareness, and perceived sagittal trunk appearance in this population. *Materials and Methods:* This cross-sectional study included 250 of 512 screened desk-based workers with CNNP, pain severity ≥4/10, and a Rapid Upper Limb Assessment (RULA) score ≥ 3/7. Physical activity, upper-extremity disability, body awareness, perceived sagittal trunk appearance, and ergonomic risk were assessed using the IPAQ-SF, QuickDASH, Body Awareness Rating Questionnaire (BARQ), KyphoTAPS, and a video-call-based RULA. *Results:* Participants reporting exercise participation had lower QuickDASH scores (*p* = 0.003, d = −0.42) and higher BARQ total scores (*p* = 0.023, d = 0.33) than non-exercisers. BARQ function and awareness scores were higher in this group (*p* = 0.004 and *p* = 0.033), while pain severity, perceived sagittal trunk appearance, and ergonomic risk were similar between groups. QuickDASH scores were positively correlated with pain severity (rho = 0.636–0.727) and negatively correlated with BARQ total scores (rho = −0.477). RULA scores showed a weak positive correlation with general neck pain (rho = 0.288) (FDR-adjusted *p* < 0.002). Exploratory adjusted analyses showed that participants reporting regular exercise participation had lower QuickDASH and higher BARQ total scores than non-exercisers in all models. Participants reporting low-frequency exercise participation had lower QuickDASH scores than non-exercisers only in Model 4. *Conclusions:* Self-reported exercise participation may be associated with lower QuickDASH scores and higher BARQ total scores in desk-based workers with CNNP and increased ergonomic risk. Because maintaining consistent exercise participation can be challenging in occupational settings, future studies should investigate feasible exercise strategies to address pain-related, functional, and perceptual outcomes among occupational populations exposed to prolonged ergonomic loading.

## 1. Introduction

In recent years, rising technological demands and digital transformation have increased both the physical and cognitive workloads of desk-based workers [1,2,3]. Prolonged sitting, low-level muscle activity, and ergonomically unfavorable postures may contribute to chronic musculoskeletal pain and functional limitations [4,5,6,7,8]. Neck pain is common among office and computer workers and may be accompanied by functional limitations, reduced quality of life, and reduced work productivity, particularly due to presenteeism [9,10,11,12]. Prolonged computer use, limited work breaks, repetitive upper-extremity movements, and higher ergonomic risk have also been associated with neck and upper-extremity symptoms in this population [9]. Moreover, neck pain in desk-based workers may coexist with shoulder and upper-extremity complaints rather than occurring as an isolated cervical symptom [9,13]. Because upper-extremity activity may aggravate neck symptoms, upper-extremity disability is a relevant functional characteristic in this population [14]. Prolonged exposure to ergonomically unfavorable working conditions may exacerbate these symptoms and functional impairments [15,16,17]. Chronic musculoskeletal pain is also understood as a multifactorial condition involving physical exposure, functional consequences, and individual perceptual characteristics, rather than pain intensity alone [17,18]. Therefore, desk-based workers exposed to increased ergonomic risk may constitute an important subgroup for examining pain-related, functional, and perceptual characteristics.

The biopsychosocial model provides a framework for considering these characteristics together. In desk-based workers, prolonged computer use and ergonomic risks represent occupational and physical exposures, whereas pain and upper-extremity disability indicate symptom severity and functional impairment. Body awareness and perceived sagittal trunk appearance may also provide information about how individuals perceive their bodies. Although these features may be related to chronic musculoskeletal pain, their specific roles in chronic non-specific neck pain (CNNP) remain poorly understood [15,17,18].

Exercise participation and physical activity levels are commonly considered modifiable factors that may be associated with musculoskeletal problems in occupational populations [19,20,21]. In desk-based workers with CNNP and increased ergonomic risk, these factors may be relevant to musculoskeletal health and functional status [19,22,23]. A recent systematic review and meta-analysis found that strengthening exercise may reduce pain and disability among office workers with chronic neck pain, although the evidence was limited by the methodological quality of the included studies [4]. Exercise participation and overall physical activity should be considered related but distinct constructs. Exercise participation refers to involvement in planned or structured exercise, whereas overall physical activity encompasses a broader range of activities, including walking, as well as moderate- and vigorous-intensity activities performed in various contexts [24]. However, maintaining regular exercise participation may be difficult for many desk-based workers due to workload, fatigue, lack of time, limited workplace facilities, and insufficient organizational support [25,26,27,28]. Examining exercise participation patterns among desk-based workers may therefore be clinically relevant, because evidence on their associations with pain, functional status, and body-related perceptual characteristics in desk-based workers with CNNP remains limited [17,29,30].

Previous studies have examined the relationships between ergonomic exposures, pain intensity, and upper-extremity disability, as well as the association between physical activity and musculoskeletal symptoms among desk-based workers exposed to increased ergonomic risk. However, these characteristics have generally been examined separately [9,31]. Evidence regarding body awareness and body-related perception is less developed, particularly in populations with chronic neck pain [15,17]. Recent findings suggest that body and neck awareness may be associated with pain, disability, and proprioceptive characteristics in individuals with CNNP; however, further studies are needed to better understand these relationships [32]. Perceived sagittal trunk appearance reflects how individuals perceive their sagittal alignment, which may differ from objectively measured posture and RULA-based ergonomic risk. As most evidence concerning this construct originates from populations with sagittal deformities, its assessment in desk-based workers with CNNP should be considered exploratory [33]. Few studies have examined exercise participation, pain, upper-extremity disability, body awareness, perceived sagittal trunk appearance, and ergonomic risk within the same sample of desk-based workers with CNNP, particularly in Türkiye [15,30,34,35,36]. Examining these characteristics together may provide a clearer understanding of their relationships with exercise participation in this population. Nonetheless, given the cross-sectional nature of the study, these relationships should not be interpreted as causal.

This study investigated the relationships among exercise participation, upper-extremity disability, pain intensity, body awareness, and perceived sagittal trunk appearance in desk-based workers with CNNP and increased ergonomic risk. In addition, this study examined correlations among pain, disability, body awareness, ergonomic risk level, perceived sagittal trunk appearance, and physical activity level to better understand these clinical features and to inform future preventive rehabilitation research for desk-based workers.

## 2. Materials and Methods

### 2.1. Study Design and Participants

This study was conducted as a cross-sectional observational study to examine the associations between exercise participation, pain-related outcomes, body awareness, and perceived sagittal trunk appearance in desk-based workers with chronic non-specific neck pain (CNNP) exposed to increased ergonomic risk. Ethical approval was obtained from the Research Ethics Committee of the Faculty of Physical Therapy and Rehabilitation at Hacettepe University, Ankara, Türkiye (FTREK25/73). All procedures adhered to the principles of the Declaration of Helsinki. All participants provided informed consent before participation.

Data were collected between May 2025 and November 2025. Participants were recruited from different occupational backgrounds, including administrative staff, engineers, healthcare professionals, and academic personnel. All participants performed desk- and computer-based tasks involving prolonged sitting and regular computer use. While no minimum daily duration of computer use was specified, eligibility required at least six months of similar desk-based occupational activity. Inclusion criteria encompassed individuals aged 20 to 65 years, with a diagnosis of CNNP from a family physician or relevant specialist, experiencing neck pain for a minimum of three months, and reporting a neck pain intensity of ≥4 on the numerical rating scale (NRS). Additional eligibility criteria included active employment for at least one year and a Rapid Upper Limb Assessment (RULA) score of ≥3, reflecting increased ergonomic risk.

Participants were excluded if they had a specific diagnosed cervical or upper-extremity musculoskeletal condition other than CNNP, neurological, cardiac, or rheumatological disorders, a history of cervical spinal surgery or traumatic injury, had received physical therapy within the previous six months, participated in competitive or professional sports, were pregnant, or regularly used antidepressant medication. Participants were recruited through study advertisements posted on social media platforms (Facebook, Instagram, and X) and via email.

Participants were evaluated in two stages. In the first stage, eligible individuals completed an online questionnaire via Google Forms after providing informed consent. The questionnaire collected demographic data, exercise participation, physical activity levels, handedness, upper-extremity symptoms, pain intensity, body awareness, and perceived sagittal trunk appearance. Based on their responses to the exercise participation question, participants were initially classified as exercisers or non-exercisers. Detailed classification procedures are described in the Exercise Participation section. The online questionnaire session lasted approximately 10 min. At the end of the questionnaire, participants provided contact information to be invited to a second-stage ergonomic assessment via video call. In the second stage, participants attended an online video call for ergonomic assessment within their usual work environment. During this call, working posture was observed for approximately 5 min, and ergonomic risk was assessed using RULA. Details of the video call procedure are provided in the RULA section.

### 2.2. Outcome Measures

The primary outcomes of this study were the QuickDASH and BARQ total scores. Secondary clinical outcomes included pain intensity and perceived sagittal trunk appearance, while the BARQ subscales were analyzed as exploratory outcomes. RULA scores served to assess ergonomic risk, and IPAQ-SF scores were used to evaluate overall physical activity.

#### 2.2.1. Demographic Data

The demographic data collected included age, sex, height, body weight, education level, and occupation. Body mass index (BMI) was calculated in kg/m^2^ using self-reported height and body weight values.

#### 2.2.2. Exercise Participation

Exercise participation was assessed using a single self-reported question in the demographic questionnaire. Participants were asked, ‘Which statement best describes your current exercise habit?’ and selected one of the following options: (1) ‘I exercise regularly’ (≥2 times per week), (2) ‘I exercise irregularly’ (up to once per week), or (3) ‘I do not exercise’. No specific recall period or minimum exercise duration was specified. Interruptions in exercise participation were not assessed separately. For the main two-group analysis, individuals choosing options one or two were classified as exercisers, while those selecting option three were classified as non-exercisers. The ≥2 times per week threshold was based on previous research [21,37,38].

For the exploratory three-group analysis, the original self-reported response categories were used: regular exercise participation, low-frequency exercise participation, and no exercise participation. The exercise participation question did not assess exercise type, intensity, or duration.

#### 2.2.3. Physical Activity Level

Physical activity levels were evaluated using the validated Turkish version of the International Physical Activity Questionnaire–Short Form (IPAQ-SF), which assesses activity over the previous seven days [39]. Total physical activity was expressed in metabolic equivalent task minutes per week (MET-min/week) [39]. The questionnaire evaluates the frequency and duration of walking (3.3 MET), moderate-intensity activity (4 MET), and vigorous-intensity activity (8 MET) performed for at least 10 min at a time, as well as sitting time [40]. The weekly duration of moderate-to-vigorous physical activity was calculated as the sum of the minutes of moderate and vigorous activity reported in the IPAQ-SF. Walking minutes were not included, and these values did not represent exercise-specific activity.

Exercise participation and physical activity level were treated as separate constructs. The exercise participation question was used only for group classification and did not include a predefined recall period or a minimum session duration. In contrast, the IPAQ-SF was used to quantify overall physical activity over the previous seven days, based on the frequency and duration of walking, moderate-intensity activity, and vigorous-intensity activity. However, the two measures were not considered fully independent because exercise participation may contribute to the IPAQ-SF total score. Because both measures were obtained during the same assessment period, their temporal order could not be determined. Therefore, IPAQ-SF could not be considered a definite confounder and may represent an overlapping behavioral measure or a possible intermediate variable.

#### 2.2.4. Ergonomic Risk Level Assessment—Rapid Upper Limb Assessment (RULA)

Ergonomic risk level was assessed using RULA, an observational method that evaluates postural loading of the neck, trunk, and upper extremities during work-related tasks. Scores range from 1 to 7, with higher scores indicating greater ergonomic risk. A score of 3 or higher indicates a need for further ergonomic evaluation and possible workplace modification [41,42,43].

Participants were asked to position the camera to the side so the assessor could view their upper extremities and feet. This positioning allowed the body segments and joint angles required for RULA scoring to be observed more clearly. Similar side views have also been used in previous video-based ergonomic assessment studies [44,45]. The assessment was conducted live and not recorded. Participants continued their usual work during the 5-min video call, and the posture observed during this task was scored. A single posture considered most representative of the participant’s usual working posture during the observation period was selected and scored to obtain the final RULA score. No standardized or worst-case posture was requested. Repetition, static posture, and force/load were confirmed verbally during the call. All assessments were performed by the same physiotherapist, who had nine years of clinical and research experience in ergonomic risk assessment. Her master’s and doctoral research also included RULA assessments. The assessor was not formally blinded to participants’ exercise status because she had access to information collected during the first stage of the study.

#### 2.2.5. Quick Disabilities of the Arm, Shoulder and Hand Questionnaire (QuickDASH)

Upper-extremity disability was evaluated using the QuickDASH, a widely recognized self-report instrument designed to assess activity limitations and functional impairments of the upper limb. The validity and reliability of the Turkish version of QuickDASH have been reported previously. The questionnaire consists of 11 items scored on a 5-point Likert scale, with higher scores indicating greater disability [46].

#### 2.2.6. Pain Intensity

General neck pain and pain experienced during the previous week were evaluated using the 0–10 Numerical Rating Scale (NRS), a widely used, one-dimensional pain-rating scale. Overall neck pain over the past three months was used as an eligibility criterion, with a score of ≥4/10 required for inclusion. Neck pain during the last week was also recorded, with scores ranging from 0 (no pain) to 10 (worst imaginable pain) [47,48].

#### 2.2.7. Body Awareness Rating Questionnaire (BARQ)

The BARQ evaluates four conceptually distinct factors: function, mood, feeling/emotion, and awareness [35,49]. Each subscale includes six items scored on a 7-point Likert scale. Subscale scores range from 6 to 42, and the total score ranges from 24 to 168. Higher scores indicate greater body awareness. The Turkish version of the BARQ has demonstrated acceptable validity and reliability [35].

#### 2.2.8. Edinburgh Handedness Inventory (EHI)

Hand dominance was assessed using the EHI, a widely used questionnaire that determines hand preference for activities such as writing, throwing, and using scissors. The validity and reliability of the Turkish version of this inventory have been demonstrated previously. Scores range from −100 to +100, with higher scores indicating stronger right-hand dominance and lower scores indicating stronger left-hand dominance [50,51].

#### 2.2.9. Kyphosis Trunk Appearance Perception Scale (KyphoTAPS)

Perceived sagittal trunk appearance was assessed using KyphoTAPS, a single-item, drawing-based tool adapted from the Trunk Appearance Perception Scale and originally developed to evaluate perceptions of sagittal trunk deformity in individuals with hyperkyphotic posture. The item includes five figures representing varying degrees of kyphosis, from 1 (normal kyphosis) to 5 (severe hyperkyphosis), with higher scores indicating increased perceived postural deformity [52]. In this study, KyphoTAPS was used to assess participants’ self-perceived sagittal trunk appearance.

### 2.3. Sample Size

Sample size estimation was performed using G*Power software, Version 3.1.9.6 (Heinrich-Heine-Universität Düsseldorf, Düsseldorf, Germany) [53] for a two-tailed bivariate correlation analysis. Based on previous observational studies reporting moderate associations among physical activity level, pain, disability, and body-awareness-related outcomes in musculoskeletal populations [35,54,55], a conservative moderate correlation of r = 0.30 was assumed with an alpha level of 0.05 and 90% statistical power, resulting in a minimum required sample size of 112 participants. Considering possible participant loss during the second-stage video assessment, the target sample size was increased by 15% to 132 participants. The calculation was based on a single correlation and therefore did not include separate groups or predictors.

### 2.4. Statistical Analysis

Statistical analyses were performed using IBM SPSS Statistics for Windows, Version 26.0 (IBM Corp., Armonk, NY, USA). All 250 participants in the final analytic sample had complete data; therefore, no missing-data imputation was required. Data were screened for missing values and distributional characteristics before the analyses. Descriptive statistics were presented as mean ± standard deviation (or median and interquartile range, as appropriate) for continuous variables and as frequency (%) for categorical variables. Internal consistency in the present sample was assessed using Cronbach’s alpha for the BARQ total score and each subscale. The two-group comparisons between exercisers and non-exercisers constituted the main analyses, whereas the three-group comparisons were considered exploratory. Normality was assessed using the Shapiro–Wilk test, and homogeneity of variance was evaluated using Levene’s test. Comparisons between exercisers and non-exercisers were performed using independent-samples *t*-tests for normally distributed continuous variables, Mann–Whitney U tests for non-normally distributed continuous variables, and chi-square tests for categorical variables. Exploratory comparisons among the three exercise-participation categories were performed using one-way ANOVA with Bonferroni post hoc correction when the homogeneity assumption was met, Welch’s ANOVA with Games–Howell post hoc comparisons when it was violated, and Kruskal–Wallis tests with Bonferroni-adjusted Mann–Whitney U tests for ordinal or non-normally distributed variables. Differences in IPAQ-SF total scores across the three exercise-participation categories were examined using the Kruskal–Wallis test with Bonferroni-adjusted pairwise comparisons. Spearman’s rank correlation analysis was used to examine relationships among physical activity level, pain, disability, body awareness, ergonomic risk, and perceived sagittal trunk appearance. Benjamini–Hochberg false discovery rate (FDR) correction was applied to the correlation analyses included in the correlation matrix. Sequential ANCOVA models were used to examine the associations of exercise participation with QuickDASH and BARQ total scores. Model 1 was adjusted for age, sex, height, and body mass index; Model 2 additionally included occupational duration, weekly working hours, and RULA score; Model 3 further included general neck pain intensity and was considered the principal adjusted model; and Model 4 additionally included total physical activity level measured using the IPAQ-SF (MET-min/week) and was treated as a sensitivity analysis. Multicollinearity in Model 4 was examined using tolerance and variance inflation factor (VIF) values in both the main two-group and exploratory three-group analyses. The assumptions of linearity, homogeneity of regression slopes, normality of residuals, and homoscedasticity were assessed and considered acceptable for all ANCOVA models. Adjusted means, mean differences, 95% confidence intervals, and Bonferroni-adjusted pairwise comparisons were reported. Effect sizes were reported as Cohen’s d or r for two-group comparisons, eta-squared (η^2^) or epsilon squared (ε^2^) for subgroup analyses, partial eta squared (η^2^_p_) for adjusted models, and Cramer’s V for categorical comparisons. Statistical significance was accepted as *p* < 0.05.

## 3. Results

**Figure 1:**
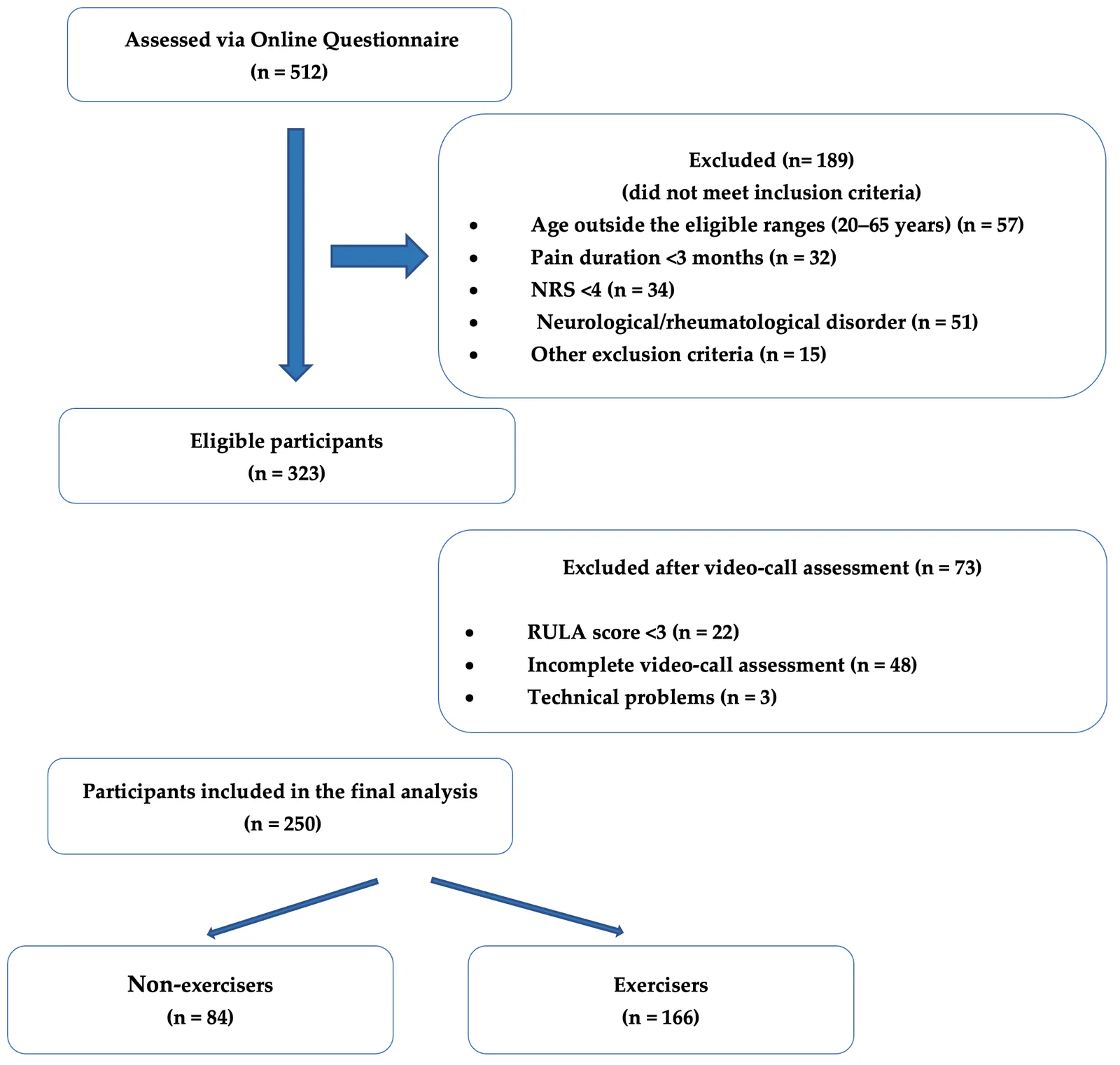
Flow diagram.

A total of 512 individuals completed the online assessment. After excluding participants who did not meet eligibility criteria or did not attend the video-call assessment, 250 participants were included in the final analyses (Figure 1). Of these, 84 were classified as non-exercisers, 92 reported low-frequency exercise participation, and 74 reported regular exercise participation. For the main two-group analysis, the latter two categories were combined as the exerciser group (n = 166). All 250 participants in the final analysis had complete data for all study variables.

Most participants held a bachelor’s degree (n = 184), followed by a master’s degree (n = 56) or a doctoral degree (n = 10). Participants came from several office-based occupational groups, including engineering and technical (n = 84), administrative and office-based (n = 67), healthcare (n = 52), and education or academic professions (n = 47).

Mean weekly moderate-to-vigorous physical activity was 158.1 ± 83.4 min among participants reporting regular exercise participation, and 98.0 ± 56.4 min among those reporting low-frequency participation. These values were calculated from the IPAQ-SF duration items for moderate- and vigorous-intensity activity and did not represent exercise-specific activity.

The two groups were comparable in age, body weight, BMI, work duration, weekly working hours, sex distribution, hand dominance, educational level, occupational distribution, and RULA scores (Table 1). However, participants reporting exercise participation were taller than non-exercisers (173.82 ± 9.93 cm vs. 170.55 ± 9.15 cm, *p* = 0.012, Cohen’s d = −0.34). Participants reporting exercise participation also had higher IPAQ-SF scores (1689.45 ± 1056.25 vs. 889.25 ± 732.04 MET-min/week, *p* < 0.001, r = 0.40) (Table 1).

Across the original three exercise-participation categories, IPAQ-SF total scores differed significantly (Kruskal–Wallis H(2) = 44.073, *p* < 0.001). Median (Q1–Q3) scores were 647.8 (460.5–1273.6) MET-min/week in non-exercisers, 1388.3 (741.3–2195.5) in participants reporting low-frequency exercise participation, and 1857.5 (950.3–2813.3) in participants reporting regular exercise participation. Bonferroni-adjusted pairwise comparisons showed higher IPAQ-SF scores in participants reporting either low-frequency or regular exercise participation than in non-exercisers (both adjusted *p* < 0.001). In contrast, the difference between the low-frequency and regular exercise-participation groups was not statistically significant (adjusted *p* = 0.075).

In the present sample, Cronbach’s alpha was 0.854 for the BARQ total score and 0.744, 0.865, 0.762, and 0.605 for the function, mood, feeling/emotion, and awareness subscales, respectively. The awareness subscale showed lower internal consistency than the other subscales.

Participants reporting exercise participation had lower QuickDASH scores than non-exercisers (18.93 ± 15.51 vs. 25.81 ± 17.97, *p* = 0.003, d = −0.42) (Table 2). They had higher BARQ total scores (100.66 ± 18.98 vs. 93.76 ± 24.03, *p* = 0.023, d = 0.33). BARQ function and awareness scores were also higher in this group (*p* = 0.004 and *p* = 0.033). No significant differences were observed in neck pain during the previous week, general neck pain, perceived sagittal trunk appearance, BARQ mood, or BARQ feeling/emotion scores.

**Table 2:** Comparison of clinical outcomes between exercisers and non-exercisers (n = 250).

Variable	Non-Exercisers Descriptive Value	Participants Reporting Exercise Participation Descriptive Value	Statistical Test	Difference Estimate (95% CI) ^1^	Effect Size	*p*
Neck pain during previous week ^a^ (0–10 NRS)	2.00 (2.00–3.00)	2.00 (1.00–3.00)	U = 6201.50; Z = −1.479	0.00 (−1.00 to 0.00)	r = 0.09	0.139
General neck pain ^b^ (0–10 NRS)	5.50 (4.88–6.00)	5.00 (4.50–6.00)	U = 6218.00; Z = −1.465	0.00 (−0.50 to 0.00)	r = 0.09	0.143
KyphoTAPS (1–5 scale)	2.00 (2.00–3.00)	2.00 (2.00–3.00)	U = 6387.00; Z = −1.176	0.00 (0.00 to 0.00)	r = 0.07	0.240
BARQ total (24–168 points)	93.76 ± 24.03	100.66 ± 18.98	Welch’s t(136.82) = −2.30	6.90 (0.95 to 12.85)	**d = 0.33**	**0.023**
BARQ Function (6–42 points)	21.64 ± 7.40	24.36 ± 6.63	t(248) = −2.94	2.71 (0.89 to 4.53)	**d = 0.39**	**0.004**
BARQ Mood (6–42 points)	16.64 ± 9.05	17.14 ± 8.48	t(248) = −0.43	0.50 (−1.79 to 2.79)	d = 0.06	0.666
BARQ Feeling/Emotion (6–42 points)	30.30 ± 8.19	32.16 ± 7.02	Welch’s t(146.06) = −1.78	1.87 (−0.20 to 3.93)	d = 0.25	0.077
BARQ Awareness (6–42 points)	25.18 ± 6.79	27.00 ± 6.10	t(248) = −2.15	1.82 (0.15 to 3.49)	**d = 0.29**	**0.033**
QuickDASH (0–100 points)	25.81 ± 17.97	18.93 ± 15.51	Welch’s t(146.89) = 2.99	−6.88 (−11.42 to −2.33)	**d = −0.42**	**0.003**

Descriptive values are presented as the median (Q1–Q3) for variables analyzed with the Mann–Whitney U test and as the mean ± standard deviation for variables analyzed with independent-samples *t*-tests. Welch’s *t*-test was used when the assumption of homogeneity of variance was violated. ^1^ Difference estimates represent Hodges–Lehmann location shifts for Mann–Whitney U analyses and mean differences for *t*-tests, with 95% confidence intervals. Effect sizes are reported as r and Cohen’s d. ^a^ Overall neck pain intensity during the previous seven days. ^b^ Overall neck pain intensity during the previous three months; this measure was used as the eligibility criterion (≥4/10). Bold values indicate statistical significance (*p* < 0.05). NRS, Numerical Rating Scale; KyphoTAPS, Kyphosis Trunk Appearance Perception Scale; BARQ, Body Awareness Rating Questionnaire; QuickDASH, Quick Disabilities of the Arm, Shoulder and Hand.

Exploratory unadjusted analyses showed significant differences in general neck pain, BARQ total, BARQ function, BARQ feeling/emotion, and QuickDASH scores. Participants reporting regular exercise participation had lower general neck pain and QuickDASH scores and higher BARQ total, function, and feeling/emotion scores than non-exercisers. QuickDASH and BARQ feeling/emotion scores also differed between the regular and low-frequency exercise-participation groups (Table S1).

Spearman correlation analyses showed that QuickDASH scores were strongly positively correlated with pain during the previous week (rho = 0.727) and moderately positively correlated with general neck pain (rho = 0.636). BARQ total scores showed moderate negative correlations with QuickDASH (rho = −0.477) and general neck pain (rho = −0.429). IPAQ-SF scores showed weak negative correlations with QuickDASH and general neck pain, while RULA scores showed a weak positive correlation with general neck pain. KyphoTAPS scores showed a weak negative correlation with BARQ total and a weak positive correlation with QuickDASH (Table 3).

In the principal adjusted model (Model 3), participants reporting exercise participation had lower QuickDASH scores and higher BARQ total scores than non-exercisers. The adjusted mean differences were −4.92 points for QuickDASH (95% CI: −8.13 to −1.72, *p* = 0.003) and 5.75 points for BARQ total (95% CI: 0.68 to 10.82, *p* = 0.026). Similar findings were observed in Model 4, which was treated as a sensitivity analysis (Table 4).

Collinearity diagnostics for Model 4 showed no evidence of problematic multicollinearity. In the main two-group analysis, tolerance values were 0.824 for exercise participation and 0.773 for IPAQ-SF, with corresponding VIF values of 1.214 and 1.293. In the exploratory three-group analysis, tolerance values were 0.683 for low-frequency exercise participation, 0.585 for regular exercise participation, and 0.761 for IPAQ-SF. The corresponding VIFs were 1.465, 1.711, and 1.314, respectively. Across both analyses, all tolerance values were ≥0.308, and all VIF values were ≤3.249.

Exploratory adjusted analyses across the three exercise participation categories showed significant differences in QuickDASH and BARQ total scores across all four models. Participants reporting regular exercise participation had lower QuickDASH scores and higher BARQ total scores than non-exercisers in all models.

Participants reporting low-frequency exercise participation did not differ from non-exercisers in the principal adjusted model (Model 3), but had lower QuickDASH scores in the sensitivity analysis (Model 4). As this difference emerged only after IPAQ-SF was added to the model, it was considered sensitive to model specification and was not interpreted as a stable exercise-frequency association. The other pairwise differences were not significant (Table S2).

## 4. Discussion

In this study, among desk-based workers with CNNP and increased ergonomic risk, self-reported exercise participation was associated with lower QuickDASH scores and higher BARQ total scores than in non-exercisers. In contrast, pain severity, perceived sagittal trunk appearance, and ergonomic risk scores were similar across groups. In the exploratory subgroup analyses, participants reporting regular exercise participation had lower QuickDASH scores and higher BARQ total scores than non-exercisers in the principal adjusted model. The QuickDASH finding for low-frequency exercise participation was observed only in the sensitivity analysis and was therefore sensitive to model specification. Because the main two-group analysis combined low-frequency and regular exercise participation, the binary findings should not be interpreted as evidence of a frequency–response or dose–response relationship.

### 4.1. Exercise Participation and Clinical Outcomes

Exercise participation was associated with lower QuickDASH scores among desk-based workers with CNNP. One possible explanation is that participants reporting regular exercise participation may have greater tolerance for repetitive upper-extremity tasks and other daily work-related activities [56,57]. This may be particularly relevant given the high prevalence of upper-extremity disability among desk-based workers (33–95%) [9]. In the present study, participants reporting exercise participation had lower QuickDASH scores than non-exercisers across all adjusted models. These associations were observed in participants exposed to increased ergonomic risk, as assessed using RULA. Previous intervention studies provide possible context for this finding. For example, Yaghoubitajani et al. [56] reported that an 8-week corrective exercise program performed three times weekly (50 min/session) optimized muscle activation patterns and improved upper-extremity capacity and neck–shoulder symptoms in office workers, supporting the potential benefits of regular exercise for occupational function. Furthermore, their 1-year follow-up study [58] showed that these functional improvements were largely maintained even after a prolonged detraining period. These studies provide context for the association between self-reported regular exercise participation and upper-extremity function observed in the present study.

Beyond exercise participation itself, exercise frequency may also be related to upper-extremity function. Amatori [59] observed fewer musculoskeletal disorders among white-collar employees who exceeded recommended physical activity levels than among those who did not meet the guidelines. Consistent with these findings, Sato et al. [60] demonstrated that improvements in physical function after a 3-month aerobic–resistance circuit training program were largely confined to participants who exercised at least twice weekly. Together, these findings may partly explain the lower QuickDASH scores observed among participants reporting regular exercise participation. In the sensitivity analysis (Model 4), participants reporting low-frequency exercise participation also had lower QuickDASH scores than non-exercisers. However, this difference was not observed in the principal adjusted model and was sensitive to model specification. The adjusted between-group difference in QuickDASH was 4.92 points in the principal adjusted model and 5.97 points in the sensitivity analysis. Although these differences were statistically significant, their magnitude was modest. In patients with neck pain, Lue et al. reported a standard error of measurement of 3.17 points and a minimal detectable change (MDC) at the 95% confidence level of 8.79 points [14]. A recent systematic review and meta-analysis of people with upper-limb musculoskeletal disorders reported a pooled MDC at the 90% confidence level of 9.03 points and a pooled minimal clinically important difference (MCID) of 11.97 points for the QuickDASH [61]. Thus, the differences observed in the present study exceeded the neck-pain-specific estimate of measurement error but remained below the reported MDC and MCID estimates. However, these thresholds were derived from longitudinal changes and cannot be directly applied to the cross-sectional between-group differences examined in the present study. In addition, the populations used to establish these thresholds differed from desk-based workers with CNNP. As no population-specific threshold is currently available, the clinical importance of the observed QuickDASH differences remains uncertain. Given the large number of desk-based workers in Turkey, evidence on exercise and musculoskeletal outcomes in this population remains limited. While Valera-Calero and Varol [55] highlighted the relevance of physical inactivity to the severity of chronic neck pain in office workers, our findings suggest that self-reported exercise participation may be related to functional status in this population.

Pain severity is influenced by multiple occupational, physical, and psychosocial factors [57]. This multifactorial nature may explain why exercise participation was not consistently related to pain outcomes in the present study [16]. The observed subgroup difference for general neck pain should therefore be interpreted cautiously. Similarly, Beneka et al. [20] observed reductions in neck and upper-back pain following a 6-week exercise program conducted three times per week among office workers and healthcare personnel. These results suggest that the relationship between exercise participation and pain may differ depending on the specific pain outcome. This may partly explain why pain-related differences in the present study were observed primarily among participants reporting regular exercise participation.

Body awareness is an important perceptual component in chronic musculoskeletal pain, as it may reflect how individuals perceive and respond to bodily signals. Movement-based activities may support body awareness by increasing attention to posture, movement quality, and bodily sensations [15,30]. Previous studies suggest that body awareness is associated with functional capacity and disability in individuals with chronic musculoskeletal conditions. Olsen et al. [62] reported that body awareness was more strongly associated with functional capacity than with pain-related outcomes. Demirel et al. [35] also reported lower body-awareness-related scores in individuals with chronic low back pain and demonstrated associations between body awareness and disability levels. In the present study, the between-group difference was most evident for the BARQ total score, which combines four conceptually distinct domains, and subgroup differences were observed only for participants reporting regular exercise participation. Exploratory findings also suggested differences in the function and feeling/emotion dimensions, whereas participants reporting low-frequency exercise participation did not differ from non-exercisers. Although participants reporting exercise participation had higher BARQ awareness scores in the main two-group comparison, this dimension did not show a clear difference across the three exercise-participation categories. The awareness subscale also showed comparatively low internal consistency in the present sample (Cronbach’s α = 0.605). This value was lower than those reported in the Turkish validity and reliability study, in which Cronbach’s alpha for the awareness subscale was 0.926 among individuals with chronic low back pain and 0.819 among healthy controls [35]. Because internal consistency is sample-dependent and the populations included in the validation study differed from the present population, this comparison should be interpreted cautiously. Nevertheless, the lower internal consistency observed in the present sample should be considered when interpreting the findings for the awareness subscale. Overall, self-reported exercise participation was associated with BARQ total and selected subscale outcomes, but the patterns across subgroup findings and exercise-participation categories were not consistent. Therefore, the higher BARQ total score should not be interpreted as a generalized improvement across all body-awareness domains. Unlike studies comparing chronic pain populations with healthy controls, all participants in the present study were desk-based workers with CNNP who were exposed to similar ergonomic demands, which may have limited between-group differences. Because both exercise participation and BARQ outcomes were self-reported, the findings should be interpreted cautiously.

Perceived sagittal trunk appearance may be influenced more by long-term occupational exposure and postural habits than by exercise participation alone [16,63]. Sanchez-Raya et al. [52] described KyphoTAPS as a visual tool for evaluating perceived sagittal trunk appearance in individuals with hyperkyphotic posture. Hijazo-Larrosa et al. [63] further reported that sagittal spinal posture in office workers was more strongly related to occupational exposure than to physical activity level. In the present study, perceived sagittal trunk appearance was not associated with exercise participation, although weak associations were observed with disability and body-awareness-related outcomes. Because all participants in the present study were exposed to increased ergonomic risk, this shared occupational loading may have limited exercise-related differences in perceived sagittal trunk appearance.

Pain, disability, and body-awareness-related outcomes are relevant clinical characteristics to consider for desk-based workers with CNNP who are exposed to increased ergonomic risk [16,31]. In the present study, functional disability showed stronger associations with pain measures and body-awareness-related outcomes than with physical activity level or perceived sagittal trunk appearance. Similar associations among disability, pain severity, and body awareness have been reported in individuals with chronic neck pain and work-related musculoskeletal disorders [32,54,64]. Although ergonomic risk showed a weak association with general neck pain, it was not significantly associated with disability, body awareness, or perceived sagittal trunk appearance [42,65]. This finding may suggest that ergonomic risk is more closely associated with pain-related experiences than with functional or perceptual outcomes in this population. Because all participants were desk-based workers with CNNP exposed to increased ergonomic risk, the relatively homogeneous occupational exposure may partly explain these findings [66].

### 4.2. Limitations

This study has some limitations. First, causal relationships cannot be established because of the cross-sectional design. Second, screening-stage data were not available for the 51 individuals who did not complete the second-stage video-call assessment, including 48 who did not attend the video call and three who experienced technical problems. Therefore, these individuals could not be compared with the final analytic sample. Possible selection bias related to completion of the video-call assessment cannot be excluded, and the direction and magnitude of this potential bias remain unknown. Third, exercise participation was assessed using a single self-reported question without a predefined recall period or minimum session duration, which may have been influenced by individual reporting. Although the IPAQ-SF provided information on physical activity intensity and duration, these data were not specific to the exercise habit used to classify participants and participants may still have differed in their exercise characteristics. In addition, the concurrent and partially overlapping assessment of exercise participation and IPAQ-SF limited our ability to determine whether total physical activity was a confounder, an overlapping exposure measure, or a possible intermediate variable. Fourth, KyphoTAPS was originally designed for individuals with thoracic hyperkyphosis and has not been specifically validated in desk-based workers with CNNP. No objective postural assessment was performed. Therefore, its findings should be interpreted as reflecting self-perceived sagittal trunk appearance rather than as an objective assessment of postural abnormalities. RULA assessments were performed by a single assessor who was not formally blinded to participants’ exercise status. Study-specific intra-rater and inter-rater reliability was also not assessed. These points should be considered when interpreting the RULA findings. The inclusion of only participants with RULA scores ≥ 3 restricted the range of ergonomic risk scores and may have limited the observed differences and associations involving RULA. Finally, although the participants shared similar desk-based working conditions, occupational tasks and ergonomic demands may have varied across professional groups. Therefore, these occupational differences may limit the generalizability of the findings to workers with substantially different occupational demands or ergonomic exposures.

### 4.3. Occupational Implications and Practical Recommendations

The present study may inform occupational rehabilitation planning for desk-based workers with CNNP exposed to increased ergonomic risk. Exercise participation was associated with lower QuickDASH scores and higher BARQ total scores, suggesting that it may be relevant when planning preventive rehabilitation strategies for desk-based workers exposed to prolonged ergonomic loading. However, maintaining consistent exercise routines may be challenging for many desk-based workers due to workload, fatigue, and time constraints. Considering the large proportion of desk-based employees in Türkiye, these findings may also be relevant to occupational rehabilitation strategies targeting Turkish desk-based workers exposed to prolonged ergonomic loading.

Based on the study findings and general occupational rehabilitation principles, the following practical recommendations may be considered: (1) assessing upper-extremity disability in desk-based workers with CNNP; (2) assessing exercise participation during routine occupational health evaluations; (3) considering body awareness and ergonomic risk alongside pain and disability; (4) encouraging practical, sustainable exercise programs, brief movement breaks, and ergonomic education tailored to workers’ symptoms, workload, and time constraints; and (5) monitoring functional outcomes and adapting rehabilitation programs to individual needs. Because this was a cross-sectional study, these recommendations should be viewed as practical suggestions rather than interventions supported by causal evidence from this study. Future intervention studies are needed to evaluate feasible exercise and rehabilitation strategies in this population.

## 5. Conclusions

In desk-based workers with CNNP who were exposed to increased ergonomic risk, self-reported exercise participation was associated with lower QuickDASH scores and higher BARQ total scores. Pain severity, perceived sagittal trunk appearance, and ergonomic risk levels were generally similar between groups. In the exploratory analyses, the most consistent differences in QuickDASH and BARQ total scores were observed between participants reporting regular exercise participation and non-exercisers, while the QuickDASH finding for participants reporting low-frequency exercise participation was sensitive to model specification. These findings suggest the potential relevance of self-reported exercise participation, particularly regular participation, for occupational musculoskeletal health in desk-based workers exposed to prolonged ergonomic loading. However, because the main two-group analysis combined low-frequency and regular exercise participation, these findings should not be interpreted as evidence of a frequency–response or dose–response relationship. Future studies should investigate not only exercise participation patterns but also the short- and long-term effects of feasible exercise approaches on symptom-related and functional outcomes in occupational settings.

## Figures and Tables

**Table 1:** Demographic, ergonomic, occupational and physical activity characteristics of participants (n = 250).

Continuous Variables	Total Sample (n = 250)	Non-Exercisers (n = 84) Mean ± SD	Participants Reporting Exercise Participation (n = 166) Mean ± SD	*p*		Effect Size (95% CI)
Age (years)	32.93 ± 7.94	31.98 ± 6.42	33.41 ± 8.58	0.178		Cohen’s d: −0.18 (−0.41, 0.08)
Height (cm)	172.72 ± 9.78	170.55 ± 9.15	173.82 ± 9.93	**0.012**		**Cohen’s d: −0.34 (−0.60, −0.07)**
Weight (kg)	72.75 ± 16.3	71.93 ± 14.91	73.17 ± 16.99	0.569		Cohen’s d: −0.08 (−0.34, 0.19)
Body Mass Index (kg/m^2^)	24.17 ± 3.85	24.58 ± 3.92	23.96 ± 3.81	0.232		Cohen’s d: 0.16 (−0.10, 0.42)
RULA (1–7 score)	4.63 ± 1.09	4.64 ± 1.09	4.62 ± 1.09	0.879		Cohen’s d: 0.02 (−0.24, 0.28)
IPAQ-SF score (MET-min/week)	1420.58 ± 1030.22 1248.65 (603.25–2247.80)	889.25 ± 732.04; 647.75 (460.50–1273.60)	1689.45 ± 1056.25; 1521.55 (801.75–2450.58)	**<0.001**		**r: 0.40** **(0.37, 0.44)**
Work duration (years)	8.56 ± 6.23	8.1 ± 5.9	8.8 ± 6.4	0.408		Cohen’s d: −0.11 (−0.37, 0.15)
Weekly working hours (hours/week)	44.84 ± 8.49	45.3 ± 8.7	44.6 ± 8.4	0.532		Cohen’s d: 0.08 (−0.18, 0.34)
Categorical Variables	Total Sample (n = 250)	Non-exercisers (n = 84)	Participants reporting exercise participation (n = 166)	χ^2^(df)	*p*	Cramér’s V
Sex (F (%)/M (%)	130 (52.0)/120 (48.0)	42 (50.0)/42 (50.0)	88 (53)/78 (47)	0.203 (1)	0.653	0.028
Hand Dominance (Left/Right)	36 (14.4)/214 (85.6)	7 (8.3)/77 (91.7)	29 (17.5)/137 (82.5)	3.777 (1)	0.052	0.123
Educational level, n (%)				0.962 (2)	0.618	0.062
Bachelor’s degree	184 (73.6)	62 (73.8)	122 (73.5)			
Master’s degree	56 (22.4)	19 (22.6)	37 (22.3)			
Doctoral degree	10 (4.0)	3 (3.6)	7 (4.2)			
Occupations n (%)				1.415 (3)	0.702	0.075
Engineering and technical	84 (33.6)	30 (35.7)	54 (32.5)			
Administrative and office-based	67 (26.8)	25 (29.8)	42 (25.3)			
Healthcare	52 (20.8)	15 (17.9)	37 (22.3)			
Education or academic	47 (18.8)	14 (16.7)	33 (19.9)			

Data are presented as mean ± SD, mean ± SD together with median (Q1–Q3), or n (%), as appropriate. Independent-samples *t*-tests were used for normally distributed continuous variables. The Mann–Whitney U test was used for IPAQ-SF, and Pearson’s chi-square tests were used for categorical variables. Cohen’s d, r and Cramer’s V are reported as effect size measures. For IPAQ-SF, r was calculated as |Z|/√N. Bold values indicate statistical significance (*p* < 0.05). BMI: body mass index; RULA: Rapid Upper Limb Assessment; IPAQ-SF: International Physical Activity Questionnaire–Short Form.

**Table 3:** Spearman correlations of selected variables with BARQ total, QuickDASH, and general neck pain (n = 250).

Variable		BARQ Total	QuickDASH	General Neck Pain ^a^
QuickDASH	rho	**−0.477**	—	—
	FDR-adjusted *p*	<0.002 **	—	—
General neck pain ^a^	rho	**−0.429**	**0.636**	—
	FDR-adjusted *p*	<0.002 **	<0.002 **	—
IPAQ-SF total, MET-min/week	rho	0.125	**−0.185**	**−0.180**
	FDR-adjusted *p*	0.061	0.005 **	0.005 **
Neck pain during the previous week ^b^	rho	**−0.357**	**0.727**	**0.449**
	FDR-adjusted *p*	<0.002 **	<0.002 **	<0.002 **
RULA score	rho	−0.081	0.066	**0.288**
	FDR-adjusted *p*	0.233	0.318	<0.002 **
KyphoTAPS	rho	**−0.236**	**0.227**	0.046
	FDR-adjusted *p*	<0.002 **	<0.002 **	0.467

Values are presented as Spearman’s rank correlation coefficients (rho) and Benjamini–Hochberg false discovery rate (FDR)-adjusted *p* values. The FDR adjustment was applied across 15 prespecified correlations. ^a^ Overall neck pain intensity during the previous three months; this measure was used as the eligibility criterion (≥4/10). ^b^ Overall neck pain intensity during the previous seven days. BARQ, Body Awareness Rating Questionnaire; IPAQ-SF, International Physical Activity Questionnaire–Short Form; RULA, Rapid Upper Limb Assessment; QuickDASH, Disabilities of the Arm, Shoulder and Hand Questionnaire–Short Form; FDR, false discovery rate. ** indicates statistically significant FDR adjusted *p*-values. Spearman’s rho coefficients are shown in bold when the corresponding FDR-adjusted *p* value is < 0.05.

**Table 4:** Adjusted association of exercise participation with QuickDASH and BARQ total score across sequentially adjusted models.

Outcome	Model	Non-Exercisers (n = 84) Adjusted Mean (95% CI)	Participants Reporting Exercise Participation (n = 166) Adjusted Mean (95% CI)	Adjusted Mean Difference ^1^ (95% CI)	F	*p*	Partial η^2^	Adjusted R^2^
QuickDASH	Model 1	25.07 (21.80–28.35)	19.53 (17.24–21.82)	−5.54 (−9.59 to −1.49)	7.26 (1, 243)	0.008 **	**0.029**	**0.212**
	Model 2	25.17 (21.94–28.41)	19.51 (17.25–21.77)	−5.67 (−9.68 to −1.66)	7.75 (1, 240)	0.006 **	**0.031**	**0.230**
	Model 3	24.59 (22.00–27.18)	19.67 (17.86–21.47)	−4.92 (−8.13 to −1.72)	9.17 (1, 239)	0.003 **	**0.037**	**0.509**
	Model 4	25.29 (22.61–27.97)	19.32 (17.49–21.15)	−5.97 (−9.35 to −2.60)	12.15 (1, 238)	0.001 **	**0.048**	**0.514**
BARQ Total	Model 1	93.98 (89.56–98.40)	100.09 (97.00–103.18)	6.11 (0.63 to 11.58)	4.83 (1, 243)	0.029 *	**0.019**	**0.096**
	Model 2	93.79 (89.39–98.19)	100.16 (97.09–103.23)	6.37 (0.93 to 11.81)	5.31 (1, 240)	0.022 *	**0.022**	**0.108**
	Model 3	94.27 (90.18–98.37)	100.03 (97.17–102.89)	5.75 (0.68 to 10.82)	4.99 (1, 239)	0.026 *	**0.020**	**0.227**
	Model 4	93.92 (89.65–98.19)	100.20 (97.28–103.12)	6.28 (0.90 to 11.67)	5.29 (1, 238)	0.022 *	**0.022**	**0.224**

^1^ Adjusted mean difference was calculated as participants reporting exercise participation minus non-exercisers. Model 1: adjusted for age, sex, height, and BMI. Model 2: Model 1 plus occupational duration, weekly working hours, and RULA score. Model 3: Model 2 plus general neck pain intensity. Model 4: Model 3 plus IPAQ-SF total score. Model 3 was considered the principal adjusted model, and Model 4 was treated as a sensitivity analysis. BARQ, Body Awareness Rating Questionnaire; BMI, body mass index; CI, confidence interval; QuickDASH, Quick Disabilities of the Arm, Shoulder and Hand; RULA, Rapid Upper Limb Assessment. * *p* < 0.05, ** *p* < 0.01.

## Data Availability

Data are available from the corresponding author upon reasonable request and are not publicly available due to ethical restrictions.

## Supplementary Materials

The following supporting information can be downloaded at https://www.mdpi.com/article/10.3390/medicina62081602/s1, Table S1: Exploratory unadjusted comparisons of clinical outcomes across self-reported exercise-participation categories; Table S2: Adjusted comparisons of QuickDASH and BARQ total scores across self-reported exercise-participation categories.

## Abbreviations

The following abbreviations are used in this manuscript:

ANOVA	Analysis of Variance
BARQ	Body Awareness Rating Questionnaire
BMI	Body Mass Index
CNNP	Chronic Non-Specific Neck Pain
EHI	Edinburgh Handedness Inventory
IPAQ-SF	International Physical Activity Questionnaire–Short Form
MET	Metabolic Equivalent Task
NRS	Numerical Rating Scale
QuickDASH	Quick Disabilities of the Arm, Shoulder and Hand Questionnaire
RULA	Rapid Upper Limb Assessment
SPSS	Statistical Package for the Social Sciences
KyphoTAPS	Kyphosis Trunk Appearance Perception Scale
