# Supplementary material for: Exercise Participation and Clinical Characteristics of Desk-Based Workers with Chronic Non-Specific Neck Pain Exposed to Increased Ergonomic Risk: A Cross-Sectional Study
Source: Medicina (Kaunas). 2026 Aug 20;62(8):1602. doi: 10.3390/medicina62081602 (PMC13515046; doi:10.3390/medicina62081602)
Supplement: Supplementary file 1 [file medicina-62-01602-s001.zip › medicina-4391333-supplementary.pdf]

Table S1 Exploratory unadjusted comparisons of clinical outcomes across self-reported exercise-participation categories.
Panel A. Descriptive values across the three exercise-participation categories.

Variable	Non-exercisers (n = 84)	Participans reporting low-frequency exercise participation (n = 92)	Participans reporting regular exercise participation (n = 74)	Statistical test	Test statistic	p	Effect size
Neck pain during the previous week (0–10 NRS)	2.00 (2.00–3.00)	2.00 (1.00–3.00)	2.00 (1.00–3.00)	Kruskal–Wallis	H(2) = 3.40	0.183	$\varepsilon^2 = 0.006$
General neck pain (0–10 NRS)	6.00 (4.25–6.00)	5.00 (4.00–6.00)	5.00 (4.00–6.00)	Kruskal–Wallis	H(2) = 6.98	**0.030***	$\varepsilon^2 = 0.020$
KyphoTAPS (1–5 scale)	2.00 (2.00–3.00)	2.00 (2.00–3.00)	2.00 (2.00–3.00)	Kruskal–Wallis	H(2) = 4.50	0.105	$\varepsilon^2 = 0.010$
BARQ Total (24–168 points)	93.76 ± 24.03	98.12 ± 19.57	103.82 ± 17.85	Welch ANOVA	F(2, 161.75) = 4.75	**0.010***	$\eta^2 = 0.036$
BARQ Function (6–42 points)	21.64 ± 7.40	23.53 ± 6.61	25.38 ± 6.55	One-way ANOVA	F(2, 247) = 5.83	**0.003****	$\eta^2 = 0.045$
BARQ Mood (6–42 points)	16.64 ± 9.05	16.80 ± 8.09	17.57 ± 8.98	One-way ANOVA	F(2, 247) = 0.251	0.778	$\eta^2 = 0.002$
BARQ Feeling/Emotion (6–42 points)	30.30 ± 8.19	30.99 ± 7.79	33.62 ± 5.66	Welch ANOVA	F(2, 163.42) = 5.60	**0.004****	$\eta^2 = 0.034$
BARQ Awareness (6–42 points)	25.18 ± 6.79	26.79 ± 6.45	27.26 ± 5.68	One-way ANOVA	F(2, 247) = 2.40	0.093	$\eta^2 = 0.019$
QuickDASH (0–100 points)	25.81 ± 17.97	21.71 ± 17.01	15.48 ± 12.70	Welch ANOVA	F(2, 163.58) = 9.51	**<0.001*****	$\eta^2 = 0.061$

Neck pain during the previous week, general neck pain, and KyphoTAPS are presented as median (Q1–Q3) and were compared using the Kruskal–Wallis test. BARQ and QuickDASH scores are presented as mean ± standard deviation. Homogeneity of variance was evaluated using Levene's test. Standard one-way ANOVA was used when the homogeneity assumption was met, whereas Welch's ANOVA was used when this assumption was violated. Epsilon-squared (ε^2) was calculated for Kruskal–Wallis tests, and eta-squared (η^2) was calculated for continuous outcomes. *$p < 0.05$; **$p < 0.01$; ***$p < 0.001$. Bold values indicate statistically significant overall group differences ($p < 0.05$). These subgroup analyses were considered exploratory.

Panel B. Pairwise comparisons following significant overall group differences.

Variable	Pairwise comparison	Pairwise method	Difference estimate (95% CI)	Raw p	Multiplicity-adjusted p
General neck pain (0–10 NRS)	Low-frequency − Non-exercisers	Mann–Whitney U	—	0.787	1.000
	Regular − Non-exercisers	Mann–Whitney U	—	0.015	**0.045***
	Regular − Low-frequency	Mann–Whitney U	—	0.030	0.090
BARQ total (24–168 points)	Low-frequency − Non-exercisers	Games–Howell	4.36 (−3.50 to 12.22)	—	0.391
	Regular − Non-exercisers	Games–Howell	10.06 (2.15 to 17.98)	—	**0.009****
	Regular − Low-frequency	Games–Howell	5.71 (−1.18 to 12.59)	—	0.125
BARQ Function (6–42 points)	Low-frequency − Non-exercisers	Bonferroni	1.89 (−0.61 to 4.39)	—	0.208
	Regular − Non-exercisers	Bonferroni	3.74 (1.10 to 6.37)	—	**0.002****
	Regular − Low-frequency	Bonferroni	1.85 (−0.74 to 4.43)	—	0.259
BARQ Feeling/Emotion (6–42 points)	Low-frequency − Non-exercisers	Games–Howell	0.69 (−2.16 to 3.55)	—	0.835
	Regular − Non-exercisers	Games–Howell	3.32 (0.70 to 5.95)	—	**0.009****
	Regular − Low-frequency	Games–Howell	2.63 (0.16 to 5.10)	—	**0.034***
QuickDASH (0–100 points)	Low-frequency − Non-exercisers	Games–Howell	−4.10 (−10.35 to 2.15)	—	0.270
	Regular − Non-exercisers	Games–Howell	−10.33 (−16.14 to −4.52)	—	**<0.001*****
	Regular − Low-frequency	Games–Howell	−6.24 (−11.69 to −0.78)	—	**0.021***

Pairwise comparisons were performed only for outcomes with a significant overall group effect. Exercise-participation categories were based on a single self-reported question. General neck pain was analyzed using Mann–Whitney U tests with Bonferroni-adjusted p values. Parametric comparisons are presented as mean differences with 95% confidence intervals. Bonferroni or Games–Howell tests were used according to variance homogeneity. Bold values indicate statistically significant pairwise differences. *p < 0.05; **p < 0.01; ***p < 0.001.

Table S2. Adjusted comparisons of QuickDASH and BARQ total scores across self-reported exercise-participation categories.
Panel A. Adjusted mean scores across the three self-reported exercise participation categories.

Outcome	Model	Non-exercisers (n = 84) adjusted mean (95% CI)	Participants reporting low-frequency exercise participation (n = 92) adjusted mean (95% CI)	Participants reporting regular exercise participation (n = 74) adjusted mean (95% CI)	F (df1, df2)	p	Partial η^2	Adjusted R^2
**QuickDASH**	**Model 1**	25.20 (21.93–28.46)	21.10 (18.04–24.16)	17.49 (13.98–20.99)	4.80 (2, 243)	**0.009****	**0.038**	**0.216**
	**Model 2**	25.29 (22.05–28.53)	20.94 (17.92–23.97)	17.63 (14.16–21.10)	4.87 (2, 240)	**0.008****	**0.039**	**0.233**
	**Model 3**	24.65 (22.06–27.24)	20.37 (17.94–22.79)	18.75 (15.96–21.53)	4.94 (2, 239)	**0.008****	**0.040**	**0.509**
	**Model 4**	25.42 (22.74–28.11)	20.20 (17.79–22.62)	18.10 (15.26–20.94)	6.69 (2, 238)	**0.001****	**0.053**	**0.515**
**BARQ total**	**Model 1**	93.80 (89.39–98.21)	97.77 (93.64–101.89)	103.12 (98.39–107.84)	3.82 (2, 243)	**0.023***	**0.030**	**0.102**
	**Model 2**	93.61 (89.22–97.99)	97.87 (93.77–101.98)	103.15 (98.44–107.85)	4.03 (2, 240)	**0.019***	**0.032**	**0.114**
	**Model 3**	94.13 (90.03–98.23)	98.35 (94.52–102.18)	102.23 (97.82–106.63)	3.34 (2, 239)	**0.037***	**0.027**	**0.229**
	**Model 4**	93.66 (89.38–97.94)	98.45 (94.60–102.29)	102.62 (98.10–107.15)	3.61 (2, 238)	**0.029***	**0.029**	**0.227**

Panel B. Bonferroni-adjusted pairwise comparisons between the three self-reported exercise participation categories.

Outcome	Model	Comparison	Adjusted mean difference (95% CI)	Bonferroni-adjusted p
**QuickDASH**	**Model 1**	Low-frequency − Non-exercisers	−4.10 (−9.55 to 1.36)	0.214
		Regular − Non-exercisers	**−7.71 (−13.74 to −1.68)**	**0.007****
		Regular − Low-frequency	−3.61 (−9.36 to 2.13)	0.392
	**Model 2**	Low-frequency − Non-exercisers	−4.35 (−9.74 to 1.05)	0.160
		Regular − Non-exercisers	**−7.66 (−13.64 to −1.68)**	**0.007****
		Regular − Low-frequency	−3.32 (−9.01 to 2.38)	0.486
	**Model 3**	Low-frequency − Non-exercisers	−4.28 (−8.60 to 0.04)	0.053
		Regular − Non-exercisers	**−5.90 (−10.70 to −1.10)**	**0.010***
		Regular − Low-frequency	−1.62 (−6.19 to 2.95)	1.000
	Model 4	Low-frequency − Non-exercisers	−5.22 (−9.66 to −0.78)	**0.015***
		Regular − Non-exercisers	−7.32 (−12.40 to −2.25)	**0.002****
		Regular − Low-frequency	−2.10 (−6.69 to 2.48)	0.808
**BARQ total**	**Model 1**	Low-frequency − Non-exercisers	3.97 (−3.39 to 11.33)	0.584
		Regular − Non-exercisers	**9.32 (1.18 to 17.45)**	**0.019***
		Regular − Low-frequency	5.35 (−2.40 to 13.10)	0.293
	**Model 2**	Low-frequency − Non-exercisers	4.27 (−3.05 to 11.59)	0.483
		Regular − Non-exercisers	**9.54 (1.44 to 17.64)**	**0.015***
		Regular − Low-frequency	5.27 (−2.45 to 13.00)	0.303
	**Model 3**	Low-frequency − Non-exercisers	4.22 (−2.61 to 11.04)	0.414
		Regular − Non-exercisers	**8.10 (0.51 to 15.68)**	**0.032***
		Regular − Low-frequency	3.88 (−3.35 to 11.11)	0.590
	Model 4	Low-frequency − Non-exercisers	4.79 (−2.28 to 11.86)	0.311
		Regular − Non-exercisers	8.97 (0.90 to 17.04)	**0.024***
		Regular − Low-frequency	4.18 (−3.12 to 11.47)	0.506

Model 1: Adjusted for age, sex, height, and BMI.
Model 2: Model 1 plus occupational duration, weekly working hours, and RULA score.
Model 3: Model 2 plus general neck pain intensity.
Model 4: Model 3 plus IPAQ-SF total score.
Model 3 was considered the principal adjusted model, and Model 4 was treated as a sensitivity analysis.
Mean differences were calculated as the first listed group minus the second listed group. Exercise-participation categories were based on a single self-reported question. Bonferroni-adjusted p values and 95% confidence intervals are reported for pairwise comparisons. BARQ, Body Awareness Rating Questionnaire; BMI, body mass index; CI, confidence interval; QuickDASH, Quick Disabilities of the Arm, Shoulder and Hand; RULA, Rapid Upper Limb Assessment. Bold values indicate statistically significant pairwise differences: *$p < 0.05$; **$p < 0.01$; ***$p < 0.001$.
